# Recent developments in the field of cachexia, sarcopenia, and muscle wasting: highlights from the 12th Cachexia Conference

**DOI:** 10.1002/jcsm.12552

**Published:** 2020-02-12

**Authors:** Nicole Ebner, Stefan D. Anker, Stephan von Haehling

**Affiliations:** ^1^ Department of Cardiology and Pneumology University of Göttingen Medical Center Göttingen Germany; ^2^ German Center for Cardiovascular Research (DZHK), partner site Göttingen Göttingen Germany; ^3^ Division of Cardiology and Metabolism, Department of Cardiology (CVK) Charité Berlin Germany; ^4^ Berlin‐Brandenburg Center for Regenerative Therapies (BCRT) Berlin Germany; ^5^ DZHK (German Centre for Cardiovascular Research), partner site Berlin, Charité Berlin Germany

**Keywords:** Cachexia, Muscle wasting, Sarcopenia

## Abstract

This article highlights preclinical and clinical studies in the field of wasting disorders that were presented at the 12th Cachexia Conference held in Berlin, Germany, in December 2019. Herein, we summarize the biological and clinical significance of different strategies including antibodies that target Fn14, Spsb 1, SAA1 treatment, ZIP14, a MuRF1 inhibitor, and new diagnostic tools like T‐cell communication targets and cut‐offs for the detection of skeletal muscle wasting. Of particular interest were the transplantation of mesenchymal stromal cells and muscle stem cell communication. Importantly, one presentation discussed the effect of metal ion transporter ZIP14 loss that reduces cancer‐induced cachexia. The potential of anti‐ZIP14 antibodies and zinc chelation as anti‐cachexia therapy may require testing in patients with cancer cachexia. Large clinical studies were presented such as RePOWER (observational study of patients with primary mitochondrial myopathy), MMPOWER (treatment with elamipretide in patients with primary mitochondrial myopathy), and ACT‐ONE as well as new mouse models like the KPP mouse. Promising treatments include rapamycin analogue treatment, anamorelin, elanapril, glucocorticoids, SAA1, antibodies that target Fn14, and a MuRF1 inhibitor. Clinical studies investigated novel approaches, including the role of exercise. It remains a fact, however, that effective treatments for cachexia and wasting disorders are urgently needed in order to improve patients' quality of life and their survival.

## Introduction

The development of preventive and therapeutic strategies against cachexia and wasting disorders, such as sarcopenia, is perceived as an urgent need by health professionals and have instigated intensive research into the pathophysiology of these syndromes.^1^, ^2^, ^3^, ^4^ This year, the conference started with an overview of muscle wasting research presented by Andrew J. Stewart Coats (UK). He impressively showed the beginning of research in this field and up to now with big hope studies like the ACT‐ONE trial. The ACT‐ONE trial is a multi‐centre, randomized, double‐blind, placebo‐controlled, dose‐finding study of the anabolic/catabolic‐transforming agent espindolol (MT‐102) that recruited subjects with cachexia and non‐small‐cell lung carcinoma (NSCLC) or colorectal cancer in Stages III and IV.^5^ Patients were randomized in a 3:1:2 fashion to one of two doses of espindolol (10 mg twice daily or 2.5 mg twice daily) or placebo and treated for 16 weeks. The results showed that only the high dose of 10 mg twice daily improves lean and fat mass after 16 weeks of treatment. Results of the functional data showed that only handgrip strength significantly increased after 16 weeks in the low‐dose and high‐dose groups of treatment, but stair climbing power and 6 min walk distance were left unaffected. This study gives rise to the question whether or not beta‐blockers can be viewed as having a class effect in the treatment of cancer cachexia. Recent facts and numbers presented by Stefan Anker (Medical University Charité Berlin, Germany) showed the failed studies in this regard testing imidapril, xilonix, and enobosarm. He stated that the biggest hope for an effective treatment is anamorelin (oral growth hormone secretagogue), which is now in phase III study. Ghrelin and ghrelin receptor agonists, such as anamorelin, showed appetite‐stimulating and growth hormone‐releasing effects and are likely to be useful for the treatment of muscle wasting. Unlike sarcopenia, cachexia is characterized by progressive weight loss affecting different body compartments, particularly muscle tissue and adipose tissue, although even bone mineral content may be affected.^6^ Muscle wasting diseases such as sarcopenia, myopathies, and cachexia are associated with the decline in differentiation of muscle cells into functional myofibres. This leads to a decrease in mobility and poor quality of life. Over the last years, the Cachexia Conference has developed into a forum for researchers from all fields of cachexia and wasting disorders. It is unique in several ways as it provides a platform for both clinicians and basic researchers to meet and discuss pathways and potential therapeutic targets as well as recent evidence from clinical trials. The 12th conference was held in Berlin, Germany, from 6 to 8 December 2019, with over 400 participants from more than 35 countries attending and over 365 abstracts being presented.

## Basic science

This year, some interesting updates on signalling pathways and small molecules were presented, especially analysis of T cells, and their potential role in this field was of interest. How T cells communicate is still unclear. Andreas Bergthaler (University of Graz, Graz, Austria) stated that cachexia is maybe a genetic program for the body component what is not a disease than more a program of inflammation answer. Maybe there is a benefit from cachexia development for the body. He stated that there are three major open questions in this regard: how do T cells communicate with adipocytes (and myocytes)? The potential role of T cells in other contexts (pathogens, cancer, mouse vs. human, etc.)? Could there be an evolutionary host‐beneficial effect of infection‐associated cachexia? Another main focus in wasting disorders research is targeting the mammalian target of rapamycin (mTOR) and mTOR complex 1 (mTORC1) pathway for age‐related disorders. In the last years, it has been shown that mTORC1 inhibition increases mass in sarcopenic muscle.^7^ The sustained mTORC1 activity in aging rat skeletal muscle results in up‐regulation of mTORC1 pathway components and concomitant loss of muscle mass. Inhibition of mTORC1 with rapalogues is not detrimental to skeletal muscle and increased mass in selected muscle groups. Rapamycin and its analogues (rapalogues) are the first generation of mTOR inhibitors, which have the same molecular scaffold but different physiochemical properties. Rapalogues are being tested in a wide spectrum of human tumours as both monotherapy and a component of combination therapy. Low‐dose rapalogue treatment reverses several protein/gene expression signatures associated with sarcopenia. The mechanistic target of mTORC1 integrates skeletal muscle's response to nutrients involving protein translation, ribosome biosynthesis, autophagy, and oxidative metabolism. In this regard, Baraldo *et al*.^8^ (University of Padua, Padua, Italy) showed that reduced mTOR signalling in muscle fibres is accompanied by the appearance of markers of fibre denervation, like the increased expression of the neural cell adhesion molecule. In order to delete raptor in adult skeletal muscle, they crossed two different transgenic lines: one expressing two loxp sites flanking exon 6 of raptor and one expressing a Cre bound to an oestrogen receptor domain under the control of a skeletal muscle promoter, human skeletal actin. This way, Cre is expressed only in skeletal muscle. Expression of Cre is not sufficient for its activation, because there is a heat shock protein complex that binds to the oestrogen receptor domain leading to a rapid degradation. After injection of tamoxifen, the heat shock protein complex is removed and Cre becomes active. Both muscle‐specific deletion of mTOR or raptor and the use of rapamycin were sufficient to induce 3–8% of neural cell adhesion molecule‐positive fibres (*P* < 0.01), muscle fibrillation, and neuromuscular junction fragmentation in 24% of examined fibres (*P* < 0.001). Mechanistically, reactivation of autophagy with the small peptide Tat‐beclin is sufficient to prevent mitochondrial dysfunction and the appearance of neural cell adhesion molecule‐positive fibres in raptor knockout muscles. This study^7^ shows that mTOR signalling in skeletal muscle fibres is critical for maintaining proper fibre innervation, preserving the neuromuscular junction structure in both the muscle fibre and the motor neuron. In addition, considering the beneficial effects of exercise in most pathologies affecting the neuromuscular junction, these findings suggest that part of these beneficial effects of exercise is through the well‐established activation of mTORC1 in skeletal muscle during and after exercise. Also, Counts and Carson^9^ (University of Tennessee Health Science Center, Memphis, TN, USA) examined the effect of feeding and fasting on skeletal muscle mTORC1 signalling in female ApcMin/+ (MIN) mice. Female C57BL/6 (B6) and MIN mice were fasted for 12 h during the light cycle. Following the 12 h fast, mice were given access to a food pellet for 1 h (B6 FED: *N* = 7; MIN FED: *N* = 8) or fasted for another hour (B6 FAST: *N* = 6; MIN FAST: *N* = 7). Blood glucose, plasma interleukin (IL)‐6, and insulin were measured. Feeding increased stomach mass, blood glucose, and circulating insulin levels in FED B6 and MIN mice. Compared with FAST mice, FED mice increased S6K1 and rpS6 phosphorylation and were positively associated to insulin levels in both B6 and MIN mice. While feeding increased 4E‐BP1 phosphorylation in B6 mice, this response was disrupted in the MIN. Additionally, insulin was associated to 4E‐BP1 phosphorylation in the B6 but not in the MIN. This showed that the feeding regulation of muscle 4E‐BP1 is disrupted by the cancer environment during the initiation of weight loss, and further investigation is warranted to determine if this is an earlier driver of cancer‐induced skeletal muscle metabolic dysfunction. In this context, James Carson (University of Tennessee Health Science Center, Memphis) showed that high running wheel activity is associated with less body weight change, higher gastrocnemius mass, and increased fat mass. He analysed the anabolic/katabolic flux in mice after 2 weeks of wheel running. He impressively showed that cachexia progression was associated with decreased dark cycle oxygen consumption (VO_2_) and respiratory exchange ratio and diminished metabolic flexibility. Muscle 4E‐BP1 phosphorylation in the light cycle is disrupted by severe cachexia and associated with decreased cage activity. Additionally, he showed that high running wheel activity was associated with less body weight change, higher gastrocnemius mass, and increased fat mass together with increased muscle 4E‐BP1 phosphorylation in the light cycle. The fasting induction of adenosine monophosphate‐activated protein kinase (AMPK) was accelerated in the MIN mouse and normalized by wheel activity. The fasting induction of forkhead box protein O (FoxO) p‐FOXO3a and p‐ULK‐1 is accelerated in the MIN mouse and normalized by wheel activity. The fasting induction of muscle RING finger 1 (MuRF‐1) but not atrogin‐1 was increased in the MIN mouse and normalized by wheel activity. The fasting induction of light chain (LC) 3B (II/I) was increased in the MIN mouse and normalized by wheel activity. Cachexia in the MIN mouse is associated with a disrupted diurnal metabolic flexibility that coincides with decreased cage activity, an accelerated fasting response in muscle involving AMPK and autophagy signalling. Increased physical activity can normalize the accelerated fasting induction of muscle AMPK signalling in MIN mice. AMPK signalling regulates accelerated activation of fasting signalling during the initiation of cancer cachexia.

Other studies buttress the view that increased FoxO signalling and the activation of the transcription factors nuclear factor kappa‐light‐chain‐enhancer of activated B cells (NF‐κB), MuRF1, and muscle atrophy F‐box (MAFbx) in skeletal muscle play major roles during cachexia onset and progression.^10^ MuRF1 and MAFbx are essentially involved in muscle atrophy development. Indeed, genes whose expression levels are commonly increased during multiple models of skeletal muscle atrophy, including cancer and sepsis, are MAFbx, MuRF1, and cathepsin, and there is evidence that each are FoxO target genes. Inducers of MuRF1 and MAFbx expression are tumour necrosis factor (TNF), IL‐6, and IL‐1, and NF‐κB appears to be the most important regulator of MuRF1 and MAFbx expression in the skeletal muscle.^6^ Lima *et al*.^11^ (University of São Paulo, São Paulo, Brazil) showed in C57BL/6 wild‐type mice and IL‐1 receptor knockout mice (B6.129S7‐il1r tm1/mx/J) that weight loss was significantly higher in wild‐type mice relative to IL‐1R^−/−^ mice after accounting for tumour mass (*P* = 0.0056). IL‐1R‐deficient mice had significantly smaller tumours than wild‐type mice by weight (*P* = 0.015) and by LLC‐GFP signal intensity (*P* = 0.020). mRNA levels of IL‐1β in the tumour were decreased in IL‐1R^−/−^ compared with wild‐type mice (*P* = 0.015). These results suggest that inhibiting the IL1 signalling axis may provide a novel target.

In this regard, Cardoso‐Filho *et al*.^12^ (Universidade Estadual de Montes Claros, Unimontes, Montes Claros, Minas Gerais, Brazil) analysed the role of resveratrol in the mice model C57BL/6. Trans‐resveratrol (3,4,5‐trans‐trihydroxystilbene) is a naturally occurring polyphenol that modulates inflammatory responses that are usually found in a number of low‐grade, systemic chronic inflammatory diseases. They impressively showed the effects of resveratrol administration on inflammatory plasma biomarkers, anthropometrical parameters, skeletal muscle mass, volume and strength, and survival of C57BL/6 mice bearing a syngeneic cutaneous melanoma model. Resveratrol was administered in concentrations of 200 and 400 mg/kg body weight and increased skeletal muscle tissue mass, volume, and muscle strength. Moreover, mice treated with resveratrol showed a higher quantity of skeletal muscle fibres and higher pro‐myogenic factors. Most of these effects promoted by resveratrol were dose dependent. Moreover, the same group analysed^13^ visceral white adipose tissue samples that were collected and submitted to morphometric and pro‐adipogenic gene expression (PPAR‐γ and SREBF1) analysis. Resveratrol administration reduced white adipose tissue relative weight, adipocyte area and number, and pro‐adipogenic gene expression in white adipose tissue. Resveratrol was shown as novel treatment and should be tested in clinical trials. Another treatment that was shown is an antibody 002 against TNF‐related weak inducer of apoptosis (TWEAK). An antibody that targets the TWEAK receptor (Fn14) has been previously shown to reverse the symptoms of cachexia in tumour‐bearing mouse models and extend the lifespan of mice by restoring their body weight. Burvenich *et al*.^14^ (Olivia Newton‐John Cancer Research Institute, Melbourne, Australia) investigated via positron emission tomography imaging the glucose changes in tumour‐bearing mouse models of cachexia, to explore whether Fn14 plays a role in the metabolic changes occurring during cancer cachexia. The 18F‐FDG positron emission tomography imaging demonstrated an increased glucose uptake over time in cachectic vs. non‐cachectic tumour‐bearing mice. This was observed both in the MEF H‐Ras V12 hFn14 model and in the C26 model. Targeting Fn14 with antibody 002 was able to prevent increased 18F‐FDG uptake in C26 tumours, but more importantly, tumours of cachectic C26 mice with high 18F‐FDG uptake showed reduced 18F‐FDG after 2 days of therapy with 002. These results demonstrate that cachexia associated with Fn14 signalling is associated with increased tumour glucose metabolism and that 18F‐FDG positron emission tomography imaging could be used to monitor patient response to anti‐Fn14 antibody 002 cachexia treatments in clinical trials. Osellame *et al*.^15^ (La Trobe University, Melbourne, VIC, Australia) developed an antibody that targets the TWEAK receptor Fn14, on the tumour surface, which is able to abrogate symptoms of cachexia in syngeneic mouse models. This anti‐Fn14 antibody prolongs life by blocking body weight loss associated with cancer cachexia. They have shown that 18F‐FDG positron emission tomography imaging links Fn14 to glucose metabolic pathways in tumours that induce cachexia. They impressively showed that 18F‐FDG positron emission tomography imaging demonstrated increased glucose uptake in cachectic vs. non‐cachectic tumour‐bearing mice, suggesting a higher glycolytic activity in tumours of cachectic mice. They suggest that cachexia is driven by specific reprogramming of tumour metabolism, with downstream signalling events responsible for distal muscle and fat loss. This increase in glycolytic activity, body weight loss, and tumour growth can be reversed with anti‐Fn14 antibody treatment.

This year, some new mice models of cancer cachexia were shown. In this regard, Talbert *et al*.^16^ (Medical University of South Carolina, USA) testing the KPC (GEMM) model of cancer cachexia. Generating the KPP mouse (KrasG12D, Ptenf/f, and Ptf1aER‐CRE). Gene signatures in muscle from C‐26/LLC models are distinct from those of pancreatic cancer patients with cachexia. Body weight and muscle wasting in KPC mice do not correlate with pancreas pathology. He has shown that the 1‐year‐old KPP mice retain the ability to exhibit cachexia. He concludes that gene signature in muscle from KPP mice is similar to pancreatic cancer patients with cachexia, but caveats remain. Additionally, he showed that growth differentiation factor 15 (GDF‐15) is required for development of pancreatic cancer KPP and GDF‐15fl/fl mice exhibit a reduction in pancreatic cancer. Moreover, KPP mice at their endpoint exhibited decreased body weight compared with their littermate controls but, importantly, had similar tibia lengths, suggesting that the lower body weights of KPP mice were not due to a failure to grow.^16^, ^17^, ^18^ The KPP mouse as an improvement over existing animal models of cachexia and anticipate that KPP mice will prove to be a useful tool in improving our understanding of the mechanisms driving tissue wasting and in translating that understanding into new anti‐cachexia therapies.^16^

In cancer cachexia, some genes are constitutively activated in target tissues, such as MuRF1 and atrogin‐1 in muscle and cell death‐inducing DFFA‐like effector A (CIDEA) in adipose tissue. Utilizing promoters from MuRF1, atrogin‐1, and CIDEA, Cao *et al*.^19^ (La Trobe University, Melbourne, Australia) have generated reporter cell lines that are capable of detecting cachexia‐inducing factors released by tumour. The promoters of genes encoding MuRF1, atrogin‐1, and CIDEA were cloned into a vector that drives the reporter genes, luciferase and GFP. Constructs were stably integrated into C2C12 myoblasts and 3T3‐L1 pre‐adipocytes, which, when differentiated into myotubes and white adipose adipocytes, express receptors for soluble cachexia‐inducing factors. These reporter cell lines can react to the stimulation of cachexia‐inducing factors. Additionally, they can also be used as a potential diagnostic tool by detecting cachectic factors in the serum or plasma of animals and patients.

Adams *et al*.^20^ (TU Dresden, Dresden, Germany) showed the effect of a recently identified MuRF1 inhibitor, MCEMBL#205, on the development of muscle atrophy/dysfunction and molecular alterations. Male C57BL/6N mice (*n* = 30) were included into the study. At an age of 6 to 8 months, the animals were randomized into the following groups (*n* = 10 in each group): (i) control group (Con): normal chow and no tumour; (ii) tumour group (Tu): inoculation with tumour cells and normal chow; and (iii) tumour group on MCEMBL#205 (Tu‐205): inoculation with tumour cells and chow with MCEMBL#205. Functional analysis revealed impairment of muscle strength, which was attenuated by MCEMBL#205. Molecular analysis showed increased expression of MuRF1, markers for oxidative stress (Nox2, UCP3, ubiquitin, and nitrotyrosine), and autophagy (LC3). In addition, actin content was decreased. These molecular effects were reversed by MCEMBL#205. They concluded that tumour development is associated with functional impairment and muscle atrophy related to increased reactive oxygen species and autophagy. MCEMBL#205 might be a future pharmacological treatment to prevent functional decline in tumour cachexia patients.

In cultivated myocytes model, Li *et al*.^21^ (Charité—Universitätsmedizin Berlin, Germany) presented the role of Spsb1 in inflammation‐induced muscle atrophy and identify the downstream targets and signalling pathways that contribute to muscle atrophy. Spsb1 overexpression significantly impaired proliferation, differentiation, and fusion of C2C12 myoblasts, which resulted in the formation of atrophied myotubes. Myogenin, a critical transcription factor regulating myogenic differentiation, was down‐regulated in Spsb1 overexpressed cells. The transcripts of Myomaker and Myomixer, which encode key factors that govern the myoblast fusion, were severely decreased by Spsb1 overexpression. A search for the molecular mechanisms revealed that the expression of TβRII was up‐regulated during differentiation and Spsb1 targeted TβRII for degradation.

As glucocorticoids exert metabolic action on muscle–liver axis, Martin *et al*.^22^ (Université Jean Monnet Saint Étienne, Saint Étienne, France) investigate the potential role of glucocorticoids on skeletal muscle and hepatic metabolisms during cancer cachexia. ApcMin/+ mice were used as cancer cachexia model. Quadriceps muscle, liver, and blood samples were removed from 13‐week‐old (beginning of cachexia) and 23‐week‐old (advanced cachexia) ApcMin/+ mice and C57Bl6/J wild‐type littermates. Corticosterone concentration was significantly increased in the serum, quadriceps muscle, and liver of 23‐week‐old ApcMin/+ vs. wild‐type mice. The transcriptional signature in quadriceps muscle and liver of 23‐week‐old ApcMin/+ mice was almost completely reproduced in mice treated with dexamethasone, a glucocorticoid analogue. Preventing skeletal muscle mass loss by myostatin gene invalidation in ApcMin/+ mice restored corticosterone levels and abolished skeletal muscle and hepatic gene reprogramming. They concluded that glucocorticoids act systematically during cancer cachexia to drive a transcriptional programme that coordinately regulates skeletal muscle wasting and hepatic metabolic reprogramming.

The acute‐phase protein serum amyloid A1 (SAA1) is increased and accumulates in muscles of intensive care unit acquired weakness patients, but its relevance was unknown. Therefore, Hahn *et al*.^23^ (Charité—Universitätsmedizin Berlin, Max Delbrück Center for Molecular Medicine in the Helmholtz Association, Berlin, Germany) performed cell‐based *in vitro* and animal‐based *in vivo* experiments. The atrophic effect of SAA1 on differentiated C2C12 murine myotubes was investigated by analysing gene expression, protein content, and the atrophy phenotype. They used the caecal ligation and puncture model to induce polymicrobial sepsis in wild‐type mice, which were treated with the IκB kinase inhibitor or vehicle. Treatment of differentiated C2C12 myotubes with recombinant SAA1 caused myotube atrophy and increased IL‐6 gene expression. These effects were mediated by toll‐like receptor‐2 (TLR2) and TLR4. SAA1 increased the activity of the transcription factor NF‐κB p65 via TLR2 and TLR4 leading to an elevation of NF‐κB‐dependent gene expression. In polymicrobial sepsis of mice, skeletal muscle mass, tissue morphology, gene, and protein expression were associated with the atrophy response. Inhibiting NF‐κB signalling by IκB kinase inhibitor increased survival, reversed the inflammation‐induced atrophy programme, and diminished skeletal muscle atrophy of septic mice. SAA1 activates the TLR2/TLR4//NF‐κB p65 signalling pathway to cause myocyte atrophy. Serum amyloid A 1 and 2 are classical apolipoproteins, proteins that are mainly produced by the liver and strongly induced in response to inflammatory cytokines. To assess the potential contribution of SAA to tissue wasting *in vitro*, Molocea *et al*.^24^ (Helmholtz Center Munich, Institute for Diabetes and Cancer, Neuherberg, Germany) treated 3T3‐L1 adipocytes and C2C12 myotubes with recombinant SAA1. While lipolysis rates remained unaffected, we found a significant reduction in myotube diameter in response to SAA1 treatment, indicative of an atrophic effect. They analysed the contribution of SAA 1/2 to cachexia development *in vivo* using a liver‐specific knock‐down approach. For this purpose, they injected an AAV–miRNA targeting SAA1/2 or an AAV–control–miRNA into C26 tumour‐bearing mice. Despite a four‐fold reduction in circulating serum levels in the SAA1/2 knock‐down group, SAA1 was still highly up‐regulated in tumour‐bearing mice and no differences were observed in cachexia progression. In order to define novel liver‐secreted factors that can potentially impact cachexia development, they integrated hepatocyte‐specific RNA sequence and serum proteome analyses of C26 cachectic animals and observed a high degree of overlap. Characterizing a panel of these hepatocyte‐secreted proteins to investigate their potential adipocyte lipolysis and myotube atrophy‐mediating properties pave the way to novel treatment strategies.

Not only triggers of muscle wasting were focused in the last years, but also the importance of exercise as treatment was analysed in many studies. Exercise counteracts cachexia, but it is unclear to which extent the exercise‐dependent mechanical stimulation of muscle *per se* plays a role in exercise beneficial effects.^25^ To study the mechanisms underlying mechanical stimulation, Coletti *et al*.^26^ (Sorbonne Université, Paris, France) analysed cultured C2C12 myotubes in the absence or in the presence of a cyclic mechanical stretching stimulus and in the absence or presence of C26 tumour‐derived factors. A high level of circulating activin is an adverse prognostic factor in cancer patients. *In vitro* results demonstrate that activin may be a direct player and not just a marker of cachexia. Hahn *et al*.^23^ showed that the mechanical stretching stimulus is sufficient to counteract the adverse tumour‐mediated effects on muscle cells, in association with an increased follistatin/activin ratio in the cell culture medium, indicating that myotubes actively release follistatin upon stretching. In addition, mechanical stretching stimulus induces IL‐4 secretion by muscle cells.^23^ Recombinant follistatin counteracts C26 tumour effects on myotubes exclusively by rescuing fusion index, while recombinant IL‐4 ameliorates fusion index, as well as the myotube size, in terms of both myotube diameter and number of nuclei per myotube. These results indicate that tumour cells negatively affect muscle cells by releasing soluble factors and that mechanical stretching stimulus is sufficient to counteract these effects, by affecting the muscle secretome with autocrine/paracrine pathways.^23^ Activin and Act‐R ligands are becoming increasingly important as triggers of muscle wasting and as pharmacological targets to treat cachexia; however, because follistatin alone is incapable to entirely block the C26‐CM effects, the development of novel activin‐targeted approaches should consider the existence of further significant tumour‐secreted factors mediating cachexia.

Medhi *et al*.^27^ (Sapienza University of Rome, Italy) investigated the role of serum response factor, a transcription factor that playing a role in muscular growth, differentiation, and regeneration—in C26‐bearing mice in the absence or presence of voluntary exercise (wheel running). A decrease in the expression of serum response factor target genes such as myoblast determination protein 1 (MyoD) occurs in C26‐bearing mice, suggesting a decrease of serum response factor transcriptional activity. These tumour effects were counteracted by wheel running and associated to the rescue of muscle mass and function. However, a minimum amount of exercise (2 km/day) is necessary to keep serum response factor levels elevated in cachexia over a threshold, which is necessary to exert beneficial effects. Serum response factor levels inversely correlate with wasting in mice, suggesting that serum response factor plays a role in maintaining body mass.^25^ Physical activity rescues serum response factor expression as well as its transcriptional activity, highlighting the importance of genetic activation induced by skeletal muscle activity for muscle rescue and homeostasis. These effects could be extended to the fibre micro‐environment, including myogenic stem cell activity.

Wang *et al*.^28^ (Columbia University, USA) impressively showed a metal ion transporter ZIP14 that was up‐regulated in cachectic muscles from five independent metastatic models as well as in patients with metastatic cancer. They showed that the cytokines TNF and transforming growth factor‐β up‐regulate ZIP14 in muscles, resulting in the accumulation of intracellular zinc. ZIP proteins have been shown to import zinc into the cytosol from the plasma membrane or intracellular organelles. ZIP14 is a member of the zinc transporter family, which is involved in zinc uptake by cells. Increased zinc in muscle cells degrades myosin heavy chain, a major determinant of muscle mass and function. They concluded that ZIP14 loss reduces cancer‐induced cachexia. The potential of anti‐ZIP14 antibodies and zinc chelation as anti‐cachexia therapy should be tested in patients with cancer cachexia. One of these important pathways is the ubiquitin–proteasome system. The ubiquitin–proteasome system plays a critical role in skeletal muscle wasting. Studies from many groups over the past years have indeed identified many components in the ubiquitin conjugating system that are induced in atrophying skeletal muscle.

The evolutionarily conserved Hippo signalling pathway is frequently deregulated in cancer. Overexpression of the transcriptional co‐activators yes‐associated protein (YAP) and transcriptional co‐activator with PDZ‐binding motif (TAZ) is associated with pro‐proliferative and anti‐apoptotic effects in liver tumour cells. Hashemolhosseini *et al*.^29^ (Friedrich‐Alexander‐Universität Erlangen‐Nürnberg, Erlangen, Germany) inquired the neuromuscular role of canonical Wnt/β‐catenin and hippo signalling pathways and their nuclear effectors β‐catenin, YAP 1, TAZ, and members of the transcription factor interacting domain, and Groucho/TLE families. Denervation caused a muscular abrogation of canonical Wnt/β‐catenin and a gain in YAP1/TAZ transcription factor interacting domain signalling activity. Transcriptional profile changes in the myogenic lineage and in response to agrin pointed at potential importance of Tle3, Tle4, Tead1, and Tead4 at the post‐synapse. Knockouts of these genes via CRISPR/Cas9 gene editing led to reduced agrin‐dependent AChR clustering and diminished synaptic gene transcription in differentiated primary muscle cells. *In silico* analysis of previously reported transcription factor interacting domain 1/4 genomic occupation sites revealed evolutionary conserved areas with potential transcription factor interacting domain‐binding motifs in important synaptic genes, which relevance was functionally confirmed by luciferase assays. Overall, these data point to a role of TLE3, TLE4, transcription factor interacting domain 1, and transcription factor interacting domain 4 in acetylcholine receptor clustering and regulating expression of synaptic genes at the neuromuscular junction.

A number of elegant models were presented in order to improve our understanding of pathways involved in the wasting process. Muscle wasting has received increasing research efforts in recent years.^30^, ^31^ Further research is warranted to investigate the role of decreased physical activity for the suppression of muscle anabolic signalling during the progression of cancer cachexia.^32^ Feeding can activate cachectic muscle mTOR and protein synthesis. Stimulated contractions can attenuate muscle wasting and alter intramuscular cachectic signalling after the initiation of cachexia. Overall, there is a deficit in acute anabolic signalling induced by contraction signalling that is more pronounced than the response to feeding.^33^

## Body composition

Different techniques to measure body composition were presented during the congress including computer tomography scan, dual‐energy X‐ray analysis and magnetic resonance imaging, D3‐creatine dilution analysis, and bio impedance analysis. In the last years, resistance training and the impact of different training modalities were more and more focused. This year, the new statement of the European Working Group on Sarcopenia in Older People was discussed (*Table* 1).

**Table 1:** The consensus definition for sarcopenia from the European Working Group on Sarcopenia in Older People and cancer cachexia^34^, ^35^, ^36^, ^37^, ^38^

Cancer cachexia		Sarcopenia
Cachexia Consensus	Weight loss of >5% in previous 6 months with three of the following criteria:	(i) Diagnosis of sarcopenia is probable with low muscle strength (ii) Diagnosis is confirmed with low muscle quantity or quality (iii) Reduced physical performance along with reduced muscle strength and muscle quality/quantity represents severe sarcopenia
Conference (2006)	(i) Reduced muscle strength	
	(ii) Fatigue	
	(iii) Anorexia	
	(iv) Low fat‐free mass	
	(v) Abnormal biochemistry (elevated IL‐6 or CRP or low albumin)	
SCRINIO Working Group	Cachexia: >10% weight loss	
	Pre‐cachexia: <10% weight loss	
International Consensus on Cancer Cachexia Classification (2011)	>5% weight loss in 6 months	
	OR	
	>2% weight and BMI < 20 kg/m^2^	
	OR sarcopenia	

BMI, body mass index; CRP, C‐reactive protein; IL‐6, interleukin‐6.

The definition of sarcopenia remains a matter of discussion, and there is no globally accepted consensus for its diagnosis. The aim of the study presented by Bachettini *et al*.^39^ (Catholic University of Pelotas, Pelotas, Brazil) was to assess the effect of sarcopenia diagnostic components on mortality, as well as to compare the associations between sarcopenia diagnosed via the 2010 and 2018 consensuses of the European Working Group on Sarcopenia in Older People (EWGSOP) and mortality. They included 1291 older adults with an average of 2.6 years of follow‐up, 88 (6.8%) participants died. Severe sarcopenia was associated with an increased risk of death compared with that in people without sarcopenia when using EWGSOP [hazard ratio (HR) 3.15, 95% confidence interval (CI): 1.44–6.90] and EWGSOP2 (HR 4.11, 95% CI: 1.88–9.00). Older adults with decreased gait speed had a 76% higher risk of dying (*P* = 0.033). There was no statistically significant association between the other sarcopenia components and mortality risk.

Silveira *et al*.^40^ (University Federal of Goiás, Brazil) presented data from a longitudinal cohort study, which data were extracted from the ‘Elderly Project/Goiania’. Mid‐arm circumference was calculated using the formula: [mid‐arm circumference − (0.314 × triceps skinfold thickness)]. They divided the mid‐arm circumference into tertiles, and the lowest tertile was set as the reference group. Cut‐off points to define low calf circumference were <34 cm for men and <33 cm for women. By using specific older adults body mass index (BMI) classification, they identified obesity status as BMI > 27.0 kg/m^2^. Of the 416 participants, 66.1% were women, mean age 70.1 ± 7.1 years. During a mean 8.5 year follow‐up period, they observed 144 all‐cause deaths (34.62% of the total cohort). Mean mid‐arm circumference was 24.4 ± 3.12, and mean calf circumference was 34.3 ± 3.4. Obesity prevalence was 49.3% (95% CI: 44.45–54.10). Men and women differed in their mid‐arm circumference (25.8 ± 2.77 vs. 23.6 ± 3.0; *P* < 0.01, respectively) but not in calf circumference measure (*P* = 0.299). Cox proportional regression analysis for all‐cause mortality in obese older adults with low mid‐arm circumference was HR = 1.89, 95% CI: 1.01–3.55, *P* = 0.016, but not associated in non‐obese (*P* = 0.127). All‐cause mortality is associated with low calf circumference in non‐obese (HR 2.22, 95% CI: 1.33–3.69; *P* = 0.001) but not in obese older adults (*P* = 0.055). These findings indicate that muscle mass by anthropometric measures has different impact in all‐cause mortality according to nutritional status. Low mid‐arm circumference in obese patients and low calf circumference in non‐obese patients are strong predictors of all‐cause mortality risk among non‐institutionalized older adults.

The role of serum creatinine/cystatin C ratio as diagnostic biomarker was shown by Tsuneda *et al*.^41^ (Toyama Machinaka Hospital, Toyama, Japan). They enrolled 444 patients with chronic illness and quantified the area of bilateral psoas muscle normalized by height [total psoas index (mm^2^/m^2^)], using computed tomography. The cut‐off values of total psoas index in Asian adults were used as 636 and 392 mm^2^/m^2^ in men and women, respectively. Ninety‐four patients were diagnosed with cachexia and showed lower SCr/CysC ratio than non‐cachectic patients (0.054 ± 0.014 vs. 0.074 ± 0.021, respectively). The lower SCr/CysC ratio predicted cachexia with 84% (69–93%) sensitivity and 72% (64–78%) specificity in men and with 68% (55–80%) sensitivity and 71% (64–77%) specificity in women [area under the curve: 0.83 (0.76–0.89) and 0.75 (0.68–0.82)], using receiver operating characteristic analysis. Cut‐off values of SCr/CysC ratio were 0.071 in men and 0.058 in women with cachexia. According to multivariate logistic regression analysis, predictors for cachexia were anorexia, fatigue, and lower SCr/CysC ratio [odds ratios: 19.8 (8.3–43.4), 2.7 (1.4–5.2), and 5.1 (2.6–10.2), respectively].

To evaluate the correlation between psoas muscle area, used as a surrogate for sarcopenia, and the Clinical Frailty Scale in a cohort of non‐cardiac surgery patients, Bicamumpaka Shema *et al*.^42^ (Université de Montréal, Montreal, QC, Canada) included 78 patients with median age of 72 years. The correlation between psoas muscle area and Clinical Frailty Scale was weak, and psoas muscle area was a better predictor of post‐operative complications than Clinical Frailty Scale. Diagnosing muscle wasting is particularly important in those patients that are not yet cachectic but suffer from pre‐cachexia, a potential early stage of cachexia. Pre‐cachectic patients should be screened particularly because this group may be the target of multimodal intervention trials; therefore, a clear, easy‐to‐use, diagnostic tool is clearly needed. Therefore, Busquets *et al*.^43^ (University of Barcelona, Barcelona, Spain) present the development of an easy‐to‐use tool (CASC‐IN) for the evaluation of pre‐cachectic cancer patients.^44^, ^45^ Using a population of 179 cancer patients, affected by a different tumour types, they showed that the frequencies observed are non‐cachectic: 58 (32.4%), pre‐cachectic: 7 (3.9%), and cachectic: 114 (63.7%). They concluded that the CASC‐IN not only permits the identification of those patients that are pre‐cachectic but also serves to discriminate those patients that are cachectic and, therefore, can be included in staging determination by means of either CASCO or MiniCASCO. Identification of pre‐cachexia and the importance to measure body composition in patients of risk are in focus of recent research. In this regard, Emanuele Marzetti (Università Cattolica del Sacro Cuore, Rome, Italy) presented the importance of assessing body composition in the critically ill patients. He showed that body wasting is highly prevalent among intensive care unit patients and is a major risk factor for negative outcomes. Changes in body composition in among intensive care unit patients can be monitored using several imaging techniques. Relevant information on body composition may be gathered from imaging exams obtained for other purposes. Moreover, he concluded that biomarkers to assess body composition changes among intensive care unit patients are highly sought after. Also, Richard F. Dunne (University of Rochester Medical Center, Rochester, NY, USA) stated that a multimarker approach may offer a more comprehensive appraisal of body composition changes in intensive care unit patients and provide hints on the underlying pathophysiology. He showed how to assess muscle strength and function in cancer cachexia objectively. As gold standard, he showed the isokinetic dynamometers–lower limb strength testing. Handgrip dynamometry correlates well with isokinetic strength testing and overall body strength. These methods are reliable, valid, and associated with relevant outcomes in cancer and cachexia. Moreover, he showed that handgrip measurement was practical with portable equipment and could be used in cachexia studies. Measures of muscle/physical performance like the stair climb power test correlate well with lower limb 1‐repetition Max test, cost nothing to administer, and can be carried out by clinic staff. The 6 min walk test measures submaximal exertion and is therefore a good test of physical performance and daily functioning. The 6 min walk test cannot directly measure muscle strength but can calculate VO_2_ max. The short physical performance battery test was validated in older adults and cancer patients and correlates well with important outcomes. He concluded that muscle weakness is a central component of cachexia but often not included in the clinical diagnosis or characterization of cachexia in cachexia trials. The handgrip strength is the most common objective strength measure that was used in cachexia research, but various other objective measures exist to capture muscle weakness and physical performance. Incorporating changes in muscle strength or physical performance into an updated cachexia definition may encourage the cachexia research community to develop a more uniform assessment. The most impressive statement in this regard was from John Morley who stated: ‘I think about motivation of patients to do exercise and I realise a fascinating of a new machine is a big motivation, while is all about having fun in life’.

## Clinical trials and new treatment targets

Growth differentiation factor 15 is a putative cancer cachexia and prognostic biomarker. Therefore, Kazemi *et al*.^46^ (University of Alberta, Edmonton, Canada) showed that in treatment‐naïve patients (*n* = 46, 64.5 ± 7.7 years old, 22 male, BMI = 26.4 ± 5.7 kg/m^2^, 19 patients >5% weight loss) with metastatic NSCLC, baseline GDF‐15 in sarcopenic patients (*n* = 25) was higher compared with nonsarcopenic patients [4.8 (3.7–8.9 ng/mL) vs. 3.2 (1.8–4.2 ng/mL), *P* < 0.001]. Moreover, they showed that elevated GDF‐15 level was associated with hyperinflammation, skeletal muscle loss, and poor overall survival in metastatic NSCLC patients.

GDF‐15, also known as MIC‐1, is a distant member of the transforming growth factor‐β superfamily and has been implicated in various biological functions, including cancer cachexia, renal and heart failure, atherosclerosis, and metabolism, to better understand the MIC‐1/GDF‐15. In this regard, Samuel Breit (St Vincent's Centre for Applied Medical Research, St Vincent's Hospital, Sydney, NSW, Australia) presented data from mice model to better understand how prolonged elevation of MIC‐1/GDF‐15 impacts animals with adiposity. GDNF‐family receptor α‐like (GFRAL), an orphan member of the GFR‐α family, is a high‐affinity receptor for GDF‐15. GFRAL binds to GDF‐15 *in vitro* and is required for the metabolic actions of GDF‐15 with respect to body weight and food intake *in vivo* in mice. They showed that mice infused with recombinant MIC‐1/GDF‐15 (0.5 μg/g body weight per day) for more than 30 days reduces much more fat in obese mice that lean and reduces food intake in obese mice. He showed that the GFRAL knock‐down increases body weight in adiposity mice. Normal and obese mice respond differently to MIC‐1/GDF‐15 with a greater weight loss in obese because of loss of fat mass only in normal mice. In obese mice, MIC‐1/GDF‐15 is highly effective in correcting the associated metabolic and inflammatory derangement. The differential effects of MIC‐1/GDF‐15 are consistent with the relative protection from cachexia afforded by obesity. The knock‐down of GFRAL has a marked metabolic effect, indicating this is the major site of action of GDF‐15.

Muscle stem cell function declines during aging; therefore, the communication in the muscle stem cell niche is a target for therapeutic intervention during aging.^47^ Jerome Feige (Nestlé Institute of Health Sciences, Lausanne, Switzerland) presented that muscle stem cells are responsible for skeletal muscle maintenance and healing. He impressively show that fibro‐adipogenic progenitors and satellite cells cross‐communicate during muscle regeneration. Aging of fibro‐adipogenic progenitors impairs their support to muscle stem cells and alters myogenic commitment of muscle stem cells. Moreover, he presented WISP1, a member of the CCN family of proteins and is a matricellular protein secreted by fibro‐adipogenic progenitors upon injury and lost with age. The effects of WISP1 on muscle stem cells are specifically mediated by fibro‐adipogenic progenitors. WISP1 seems a novel pharmacological candidate to promote myogenic commitment in human Pax7+ muscle cells. CD8+ effector memory T cells accumulate in severely injured muscles and negatively affect muscle healing. In this regard, Sven Geißler (Charité University Hospital, Center for Regenerative Therapies, Berlin, Germany) presented basic principles and new insights in cell therapy of muscle injuries. Beneficial effects of mesenchymal stromal cells transplantation on muscle repair are partly due to modulation of local levels of CD8+ T cells. Paracrine function of mesenchymal stromal cells results in improvement of the function of muscle progenitor cells and modulates injury responses through paracrine signalling. The relevance of the adaptive immunity for successful muscle repair and how it is altered by local transplantation of autologous bone marrow muscle progenitor cells was presented. He showed in mice model that the transplantation of mesenchymal stromal cells improves muscle healing and correlates with locally reduced levels of CD3+ CD8+ effector memory T cells. CD8+ effector memory T cells accumulate in severely injured muscles and negatively affect muscle healing. The beneficial effects of mesenchymal stromal cells transplantation on muscle repair are partly due to modulation of local levels of CD8+ T cells. (Local) reduction of conventional T‐cell levels and concurrent enrichment of regulatory T cells is a promising therapeutic strategy to further improve muscle repair. In this regard, Winkler *et al*.^48^ (Charité University Berlin, Berlin, Germany) presented data from the HIPGEN study, which is now in Phase III. This randomized, double‐blind, placebo‐controlled study addresses surgical trauma‐related muscle injuries using local intraoperative application of allogeneic placenta‐derived, mesenchymal‐like adherent cells, using hip arthroplasty as a standardized injury model, because of the high regenerative and immunomodulatory potency of this cell type. The primary outcome was changes in the short physical performance battery test. They showed an improvement in maximal isometric contraction force in the treated abductor muscles of the low‐dose group compared with the placebo group after 26 weeks (*P* = 0.007). This first successful use of an allogeneic cell therapeutic approach in patients with skeletal muscle injury suggested that immunomodulation has a significant impact on regeneration and mediates at least partly the mode of action. Given that both endogenous regeneration capacity and immune cell function are related to age, this study will be conducted on skeletal muscle myoblasts and immune cells from patients and age‐matched healthy subjects.

Maurizio Muscaritoli (Università de Rome, Italy) showed data from an explorative study in 1853 cancer patients. The result shows that no genetic effect was significantly associated with appetite loss neither in the co‐dominant, dominant, and additive nor in the recessive model. He presented results of the prevalence of malnutrition study PreMIO. Malnutrition was defined as Mini Nutritional Assessment score <17 (*n* = 1952). Among cancer site groups, malnutrition stages significantly increase with stage of cancer (*P* < 0.001). They showed that decreased nutrient intake, weight loss, and malnutrition are highly prevalent at all cancer stages. More than 20% of cancer patients die for the direct or indirect consequences of cachexia. Nutrition therapy does not affect tumour growth, and changes in body composition and depletion of muscle mass may increase the risk of treatment toxicity. He stated that an early impairment of appetite and food intake in newly diagnosed cancer patients should be performed. The timely appropriated nutritional interventions may cost‐effectively improve outcome in cancer patients.

Kalantar‐Zadeh *et al*. (University of California Irvine, CA, USA) stated that cachexia always includes sarcopenia and protein‐energy wasting, but sarcopenia can happen without cachexia. The dialysis procedure is a perfect example of the integration of undernutrition and catabolism and how it leads to protein‐energy wasting, a single pathological entity. He summarized how cytokine activation, the increased muscle breakdown induced by dialysis, and reduced protein and energy intake lead to protein‐energy wasting. The dialysis procedure itself leads to increased inflammation with production of acute‐phase reactants, albumin, fibrinogen, and C‐reactive protein. The state of undernutrition is associated with a depleted amino acid pool, amino acid loss into dialysate, and the synthesis of acute‐phase proteins, limited substrate availability for muscle protein synthesis, and other anabolic processes. Undernutrition‐induced hypoalbuminaemia further aggravates this situation. The body therefore catabolizes muscle and tissue protein to release amino acids in plasma to maintain the amino acid pool. The muscle reacts to catabolism by activating local cytokine production. Thus, the dialysis procedure results in an increased inflammatory response that perpetuates the muscle catabolic effect in a vicious circle. Progressive secondary sarcopenia is very common in chronic kidney disease and dialysis patients and is the hallmark of protein‐energy wasting. Whereas adequate protein intake and amino acid is needed to correct/prevent sarcopenia, higher protein intake may compromise kidney health and worsen disease progression by virtue of increasing intraglomerular pressure (hyperfiltration hypothesis).

Caan (Kaiser Permanente Division of Research, CA, USA) gives an overview about the data from exercise training in cancer patients. She showed that resistance training can ameliorate the negative effects cancer has on muscle mass and improve side effects associated with chemotherapy.^49^ She gives an overview about the existing exercise training studies in the field of cancer, for example, the breast cancer trials (OptiTrain trial, PACES trial, and START trial). She showed that resistance or aerobic exercise does not improve dose reductions but resistance exercise prevents hospitalization (OptiTrain trial). Resistance training results in a stronger grip strength (PACES trial) and improves lean mass and chemotherapy completion rates (START trial).

‘Safety and Efficacy of Bimagrumab in Community‐dwelling Older Adults with Sarcopenia’ was presented by Laurent *et al*. (Novartis Institutes for BioMedical Research, Basel, Switzerland). Bimagrumab is a humanized monoclonal antibody binding to the ActRII, inhibiting downstream phosphorylation of Smad 2/3 and preventing the activity of myostatin that inhibits myogenesis. To determine the effect of 24 weeks of bimagrumab treatment on patient physical function (short physical performance battery test, 6 min walk distance, and gait speed) and skeletal muscle mass and strength on older adults with sarcopenia, they included 180 participants. Mean age was 80 years, and a total of 113 participants were in the bimagrumab arm and 67 participants receive placebo. Interestingly, the primary endpoint (change of short physical performance battery test score) was not significantly different between the groups. They found no additional benefit of bimagrumab on short physical performance battery test, gait speed, or 6 min walk distance over background therapy. But bimagrumab group showed a significant increase of 2 kg (7%) in lean body mass compared with no relevant change in the placebo group.

‘Results of RePOWER: A Global Prospective Observational Study of Patients with Primary Mitochondrial Myopathy’ was presented by Michelangelo Mancuso (University of Pisa, Pisa, Italy). RePOWER is designed to evaluate the genotypes and phenotypes identified in patients with primary mitochondrial myopathies and the regional differences in how patients with primary mitochondrial myopathies are managed. RePOWER also identified potential participants for MMPOWER‐3 study. MMPOWER is a multi‐centre, randomized, double‐blind, placebo‐controlled study, which included patients between 16 and 65 years of age with symptoms of mitochondrial myopathy and genetically confirmed mitochondrial disease. Primary mitochondrial myopathies are generally defined disorders leading to defects of oxidative phosphorylation affecting predominantly skeletal muscle. Secondary involvement of mitochondria was frequently observed in multiple neuromuscular diseases. The physiological functions of elamipretide^50^, ^51^ are the restoration of adenosine triphosphate production together with decreased reactive oxygen species emission and electron carriers back together with an higher membrane curvature resulting in normalized cardiolipin content. In the MMPOWER study, 30 patients in the elamipretide dosing groups (*n* = 9 with 0.01 mg/kg/h elamipretide, *n* = 9 with 0.1 mg/kg/h elamipretide, and *n* = 9 with 0.25 mg/kg/h elamipretide) compared with 30 patients in placebo group were included. The primary endpoint of distance covered during the 6 min walk test at Day 5 was significantly higher in high‐dose group (0.25 mg/kg/h) compared with the low‐dose and placebo groups. So they started the Phase III of the randomized, double‐blind, parallel‐group, placebo‐controlled trial to evaluate the efficacy and safety of daily subcutaneous injections of elamipretide in subjects with primary mitochondrial myopathy followed by an open‐label treatment extension. In this regard, Michelangelo Mancuso presented the positive effect of elamipretide on efficacy endpoints in Phase III. A total of 202 patients were recruited and receive for 6 months either 40 mg elimipride or placebo. In Part II, all patients receive 40 mg elamipretide. Moreover, he gives an overview to the ongoing studies in this field; for example, a phase I study of the safety of a new agent REN001 in patients with primary mitochondrial myopathy was shown. Also, a phase III, double‐blind, 9 month, randomized, placebo‐controlled, multi‐centre, parallel‐design study to evaluate the efficacy and long‐term safety of sonlicromanol in subjects with a genetically confirmed mitochondrial DNA tRNALeu (UUR) m.3243A>G mutation followed by an open‐label extension study was shown. ‘A Feasibility Study of Bezafibrate in Mitochondrial Myopathy’ was also named, but we are waiting for all these results. Interestingly from the literature, elamipretide/bendavia was also tested in zebrafish lateral cell lines as a novel antioxidant, on gentamicin‐induced hair cell damage.^52^ The treatment of bendavia exhibited dose‐dependent protection against gentamicin in both acute and chronic exposure. They found that bendavia at 150 μm conferred optimal protection from either acute or chronic exposure with ototoxin. Bendavia reduced uptake of fluorescent‐tagged gentamicin via mechanoelectrical transduction channels, suggesting its protective effects may be partially due to decreasing ototoxic molecule uptake. The intracellular death pathways inhibition triggered by gentamicin might be also included as no blockage of gentamicin was observed. These data suggest that bendavia represents a novel otoprotective drug that might provide a therapeutic alternative for patients receiving aminoglycoside treatment.^53^

## Conclusions

Prospective large studies are required in the field of cachexia, sarcopenia, and muscle wasting. From basic science, new therapeutic targets were shown including antibody that targets Fn14, MuRF1 inhibitor, Spsb 1, miRNAs, ZIP14, SAA1, and GDF‐15. The role of the muscle stem cells and mesenchymal stromal cell transplantation in the therapeutic management of muscle wasting and cachexia and the potential treatment targets were presented and discussed. Nevertheless, the definition of cachexia and sarcopenia as well as effective screening tools are of special interest. Big randomized studies were presented such as PreMio, HIPGEN, RePOWER, and MMPOWER‐3. Effective treatments were presented including MuRF 1 inhibitor, glucocorticoids, bimagrumab, urothelin A, rapalogue treatment, and elamipretide.

## Conflict of interest

None declared.
